# Gigantomastia: Advancing a Preference Score System to Enhance Care Quality and Life Standards

**DOI:** 10.1007/s00266-025-04831-x

**Published:** 2025-04-03

**Authors:** Cristina Melero-Fernández, Ana Belén Martínez-Martínez

**Affiliations:** 1 Breast Unit, Lozano Blesa University Hospital, Zaragoza, Spain; 2 Department of Nursing and Physiatry, Health Sciences Faculty, University of Zaragoza, C/Domingo Miral, s/n., 50.009, Zaragoza, Spain; 3 Institute for Health Research Aragón, Zaragoza, Spain

**Keywords:** Macromastia, Gigantomastia, Surgical waiting list, Preference score, Reduction mammoplasty, Patient classification systems

## Abstract

**Background:**

This study introduces a new Gigantomastia Preference Score (GPS) to prioritize surgical candidates based on clinical severity and quality of life impact.

**Methods:**

This retrospective study applied the newly developed GPS to the existing surgery waiting list of 213 patients at our center. The GPS was developed from evidence-based clinical practice indicators and a thorough literature review, selecting nine variables. The GPS was then used to reorder the waiting list, and comparisons were made between the original and reordered lists.

**Results:**

Implementation of the GPS significantly reordered the waiting list. The Spearman's Rank Correlation coefficient between the original and new rankings was 0.5679 (*p*-value = 1.38e-19), indicating a moderate to strong positive correlation. The Wilcoxon Signed-Rank Test yielded a statistic of 3485.0 (*p*-value = 8.44e-21), showing significant changes in patient positions. ANOVA results highlighted significant differences in BMI and largest breast weight across priority levels. Chi-Square tests revealed significant associations between priority levels and Trauma and Psychiatry reports.

**Conclusions:**

The GPS significantly improved the prioritization of patients with gigantomastia by incorporating multiple clinically relevant factors. This led to a more equitable and informed ordering of the surgical waiting list, potentially improving patient outcomes and optimizing healthcare resource allocation.

**Level of Evidence IV:**

This journal requires that authors assign a level of evidence to each article. For a full description of these Evidence-Based Medicine ratings, please refer to the Table of Contents or the online Instructions to Authors  www.springer.com/00266.

## Introduction

Gigantomastia, characterized by excessive breast growth, has an unknown etiology but may relate to gestational state or genetic predisposition, leading to hormonal excess and breast gland hypersensitivity [1, 2]. Known as macromastia, it is a benign condition not considered a medical priority, despite its disabling nature [3]. Physical symptoms include neck and back pain from breast weight, cervical and lumbar lordosis, thoracic kyphosis, shoulder grooves, chronic headache, and reduced areolar sensitivity [4–6]. Skin issues such as chronic eczema, intertrigo, and infections are common [7]. Psychologically, patients may experience anxiety, depression, irritability, sexual anxiety, and decreased self-esteem, impacting their quality of life [8–10].

Surgery is the gold standard treatment, with the inverted T technique being most accepted [11]. Endocrine or nutritional treatments may also be considered, as many patients have associated obesity [12].

This study aims to develop a new Gigantomastia Preference Score (GPS) to prioritize surgical candidates more effectively, reducing wait times and improving quality of life. Current surgical waiting lists often don't consider symptom severity or quality of life impact. The GPS addresses this by providing a more equitable methodology.

In many public health systems, such as those in Canada, the United Kingdom, and Australia, patients face long waiting lists due to high demand and limited resources [13]. In Spain, long surgical waiting lists are common, especially for non-life-threatening conditions like gigantomastia. In contrast, in countries like the United States with predominantly private healthcare systems, surgical wait times are generally shorter, but insurance coverage criteria can still result in delays [14]. Thus, GPS can help by reducing post-operative complications through better prioritization based on clinical need.

## Materials and Methods

### Patient Selection Criteria

Patients diagnosed with gigantomastia were included in the waiting list from March 2013 to June 2022, totaling 213 women. Inclusion criteria were breast growth of over 1.5 kg per breast, presence of symptoms, a mammogram, a breast ultrasound, and reports from the Traumatology and Psychiatry services assessing musculoskeletal pathology and psycho-emotional state affectation respectively.

### Data Management

Data were extracted from the surgical waiting list registry at our center. No access to individual medical records was required, and therefore, patient consent was not needed.

### GPS Components

The GPS was developed through an analysis of clinical practice indicators and a thorough review of the literature. Nine prioritization variables were selected for their relevance to clinical outcomes and patient well-being: age, BMI, breast weight, modified BREAST-Q test, lesions in the inframammary fold, physical activity at work, smoking habit, and medical reports from Trauma and Psychiatry services. These variables, which were already recorded in the patient registry and included in the inclusion criteria, were chosen to leverage their influence on both pathology and surgery by assigning levels within them to improve the process. Additionally, the waiting time variable was included to prevent minor patients from becoming urgent due to prolonged waiting times. Each component was weighted equally to create a more adaptable and universal protocol, suitable for the varying characteristics and resources of different centers.

For each of these variables, three prioritization levels (high/medium/low) were established. Below, we provide the scientific evidence supporting their inclusion as prioritization criteria, along with the values that define their categorization into each prioritization level. Finally, we present the GPS, which is the result of summing all these variables.

#### Age

Age is generally considered an independent predictor of complications in most surgical procedures. In plastic surgery, while it may not be completely independent, the frequency of complications increases with age, becoming critical over 80 years [15, 16]. Additionally, some studies [17, 18] reveal an increase in post-surgical complications, such as infection or wound healing issues, between 40 and 50 years, becoming more pronounced over 50.

Given the established relationship between age and the risk of surgical complications, we established the following three prioritization levels:

- High: 18–40 years
- Medium: 41–65 years
- Low: >65 years

#### Body Mass Index (BMI)

Body mass index (BMI) is a measure used to classify the degree of obesity. According to the World Health Organization (WHO), a BMI (kg/m^2^) of 30 or higher is considered obesity, 25–29.99 is overweight, 18.5–24.99 is normal weight, and below 18.5 is underweight.

Obesity is a known risk factor for various types of surgery. In the case of mammoplasty, a higher BMI is associated with an increased rate of post-surgical complications [19]. Zhang et al. [20], in their meta-analysis, identified reduced vascularity of adipose tissue, which predisposes patients to infections, as the main cause of postoperative complications.

However, an increased BMI is inherently associated with macromastia, as increased breast weight contributes significantly to overall weight gain. Güemes et al. [21] noted the difficulty in distinguishing symptoms caused by obesity from those caused by macromastia itself. In their study, they compared outcomes after mammaplasty between women with obesity and those without, concluding that both groups experienced significant improvements in quality of life, and highlighting that breast reduction surgery typically has a low rate of complications.

Despite this, increased BMI is still considered an important predictor of complications in surgical procedures [20]. Therefore, we followed the WHO obesity classification to set the prioritization levels, assigning higher scores to patients with lower BMI values due to their association with fewer complications:

- High: BMI < 25 (Underweight and normal weight)
- Medium: BMI = 25 to < 30 (Overweight)
- Low: BMI ≥ 30 (Obesity)

It is crucial to address the issue of restricting patients with obesity from surgery, as obesity is an inherent aspect of their condition. The balance between the risks and benefits of surgery for these patients must be carefully considered. To mitigate the risks while ensuring access to necessary surgical interventions, women with a BMI over 30 will be referred to consultations with nutritionists or endocrinologists to participate in weight loss programs prior to surgery.

#### Largest Breast

Gigantomastia is defined as excessive breast growth of more than 1.5 kg per breast [22]. The weight of the breasts is directly associated with a variety of symptoms in the waist and back, due to the load exerted on the vertebrae, intervertebral discs, and joints.

Breast reduction surgery has been shown to alleviate symptoms such as kyphosis, lordosis, back pain, and postural alterations [23], thereby improving the quality of life for these patients. In fact, a direct relationship has been observed between breast weight and symptomatology, with greater resection volumes leading to more significant symptom relief [24].

Additionally, the increase in breast volume during hormonal changes is related to a higher risk of breast cancer [25], so timely surgery can help prevent such complications.

Based on these findings, and considering that the grades of gigantomastia are not well defined, we have established the following prioritization levels for patients:

- High: > 3000 g per breast
- Medium: 2000–3000 g per breast
- Low: 1500–2000 g per breast

#### Modified BREAST-Q Test

The BREAST-Q scale^®^ is a Patient Reported Outcome Measure (PROM) that quantifies the impact of cosmetic and reconstructive breast surgery on patients before and after surgery [26]. It aims to assess both health-related quality of life (including physical, psychosocial, and sexual well-being) and patient satisfaction (including final outcome and care provided).

It consists of several scales from which clinicians can choose the most pertinent to their practice, such as reduction, augmentation, mastopexy, mastectomy, reconstruction, or breast conservation. Each scale comprises several questions arranged in a clinically relevant hierarchy, producing an independent score.

At our hospital, we use the BREAST-Q^TM^ Reduction/Mastopexy module (Spanish version) [27]. This version consists of four blocks, each generating an independent score. Notably, the scoring for the first three blocks is inverse compared to the last block. In the first three blocks, a lower score indicates greater severity, while in the last block, a higher score indicates greater severity, which can be confusing when interpreting the results. Additionally, in block 3, the "not applicable" item has no assigned score, which can distort the overall score.

Therefore, it was decided to modify the BREAST-Q test to have a uniform scoring criterion. All scales now score in the same way (the lower the score, the greater the severity), and the "not applicable" response was removed. Table 1 show the modified blocks of the BREAST-Q^TM^ Reduction/Mastopexy module. After modifying the blocks of interest, the final scale score is calculated by summing the resulting scores for each block (Table 2).

**Table 1:** **a** BREAST-Q^TM^ Reduction/Mastopexy module (block 3 modified). **b** BREAST-Q^TM^ Reduction/Mastopexy module (block 4 modified)

In the last 2 weeks how often have you presented:	Never	Sometimes	Part of the time	Most of the time	Always
1. Comfortable/relaxed during sex?	1	2	3	4	5
2. Sexually confident?	1	2	3	4	5
3. Satisfied with your sex life?	1	2	3	4	5
4. Sexually attractive when clothed?	1	2	3	4	5
5. Sexy when naked?	1	2	3	4	5
Total score	5	10	15	20	25

In the last 2 weeks how often have you presented:	Always	Most of the time	Part of the time	Sometimes	Never
1. Headache?	1	2	3	4	5
2. Pain in the breast area?	1	2	3	4	5
3. Lack of energy?	1	2	3	4	5
4. Difficulty doing intense exercise? (Ex: running)	1	2	3	4	5
5. Have you ever felt physically unbalanced?	1	2	3	4	5
6. Shoulder pain?	1	2	3	4	5
7. Difficulty sleeping due to breast discomfort?	1	2	3	4	5
8. Neck pain?	1	2	3	4	5
9. Erosions or painful marks on your shoulders due to bra straps?	1	2	3	4	5
10. Have you ever felt physically uncomfortable?	1	2	3	4	5
11. Redness under your breasts?	1	2	3	4	5
12. Back pain?	1	2	3	4	5
13. Arm pain?	1	2	3	4	5
14. Pain/tingling in your hands due to the size of your breasts?	1	2	3	4	5
Total score	14	28	42	56	70

**Table 2:** BREAST-Q^TM^ Reduction/Mastopexy module (final score)

Blocks	Minimum score	Maximum score
1. Block 1 (11 items; Likert scale from 1 to 4)	11	44
2. Block 2 (9 items; Likert scale from 1 to 5)	9	45
3. Block 3 (5 items; Likert scale from 1 to 5)	5	25
4. Block 4 (14 items; Likert scale from 1 to 5)	14	70
Total score	39	184

As can be seen, the minimum BREAST-Q score is 39 points, corresponding to the patient with the highest severity, and the maximum score is 184 points, corresponding to the patient with the lowest severity.

This modification establishes an equitable range of scores that categorizes patients into three priority levels:

- High: 39–87 points
- Medium: 88–136 points
- Low: 137–184 points.

#### Lesions in the Inframammary Fold (IMF)

The deterioration of the skin integrity in the IMF is caused by the friction from the breast and the humidity generated by sweat [28]. Among the most prevalent symptoms are itching and pain, which are commonly associated with moisture-induced skin damage. Intertrigo is one of the most common conditions in patients with gigantomastia [29], significantly diminishing their quality of life.

Breast volume resection in patients with gigantomastia has been shown to alleviate a wide range of symptoms, including those affecting the IMF [30]. Therefore, considering the frequency of these lesions as a criterion for patient prioritization is appropriate:

- High: Usual
- Medium: Occasional
- Low: Never

#### Physical Activity at Work

Symptomatic gigantomastia involves a variety of complaints that negatively impact the physical performance of affected women. Back, neck, and shoulder pain are among the disabling factors that hinder working or seeking employment [31]. Additionally, pulmonary function is compromised, leading to decreased inspiratory capacity and maximum voluntary ventilation [32].

Therefore, a higher score is given to women whose jobs require more physical effort, with the levels defined as follows:

- High: Intense physical activity at work
- Medium: Moderate physical activity at work
- Low: Low physical activity at work

#### Smoking Habit

Smoking is among the most frequent causes of complications in patients undergoing mammoplasty [33]. Nicotine induces vasoconstriction, which is linked to a higher risk of infections, impaired wound healing, and even necrosis of the nipple-areola complex. Therefore, it is strongly recommended to stop smoking at least 4 weeks before and after surgery [34].

Although there is no consensus on the classification of occasional smokers, consuming between 1 and 10 cigarettes per day seems to be the most widely accepted definition [35]. Hence, the three proposed prioritization levels are:

- High: Non-smoker (0 cigarettes per day)
- Medium: Occasional smoker (1–10 cigarettes per day)
- Low: Smoker (>11 cigarettes per day)

Smokers who wish to increase their priority level on the surgical waiting list will be included in smoking cessation programs if they desire.

#### Medical Report from Trauma and Psychiatry Service

For inclusion on the gigantomastia waiting list, it is mandatory to present reports from trauma and psychiatry specialists indicating the presence of musculoskeletal and psycho-social affectation. These reports are crucial as this condition can lead to significant physical and psychological issues, including low self-esteem and depression [32].

Currently, the degree of affectation is not specified in these reports, which limits the ability to prioritize patients effectively. In countries such as Canada, New Zealand, and South Africa, patient prioritization on waiting lists is based on various medical criteria [36, 37]. Therefore, we proposed that trauma and psychiatry specialists indicated the severity of the condition in their reports, assigning each patient to a priority level (high/medium/low) based on their clinical assessment.

#### Waiting Time

Since 2003, the Spanish Ministry of Health has annually reported on the health system, including surgical waiting lists. The 2022 report [38] states the average waiting time for non-urgent surgery was 113 days, with Plastic Surgery having the longest wait at 226 days. Excessive delays increase morbidity and mortality and distort patient data. To mitigate this, the Ministry recommends waiting times for elective surgery to be less than 6 months and not exceed 1 year. Priority levels are based on these guidelines:

- High: > 1 year
- Medium: 6–12 months
- Low: < 6 months

### Gigantomastia Preference Score (GPS)

**Fig. 1:**
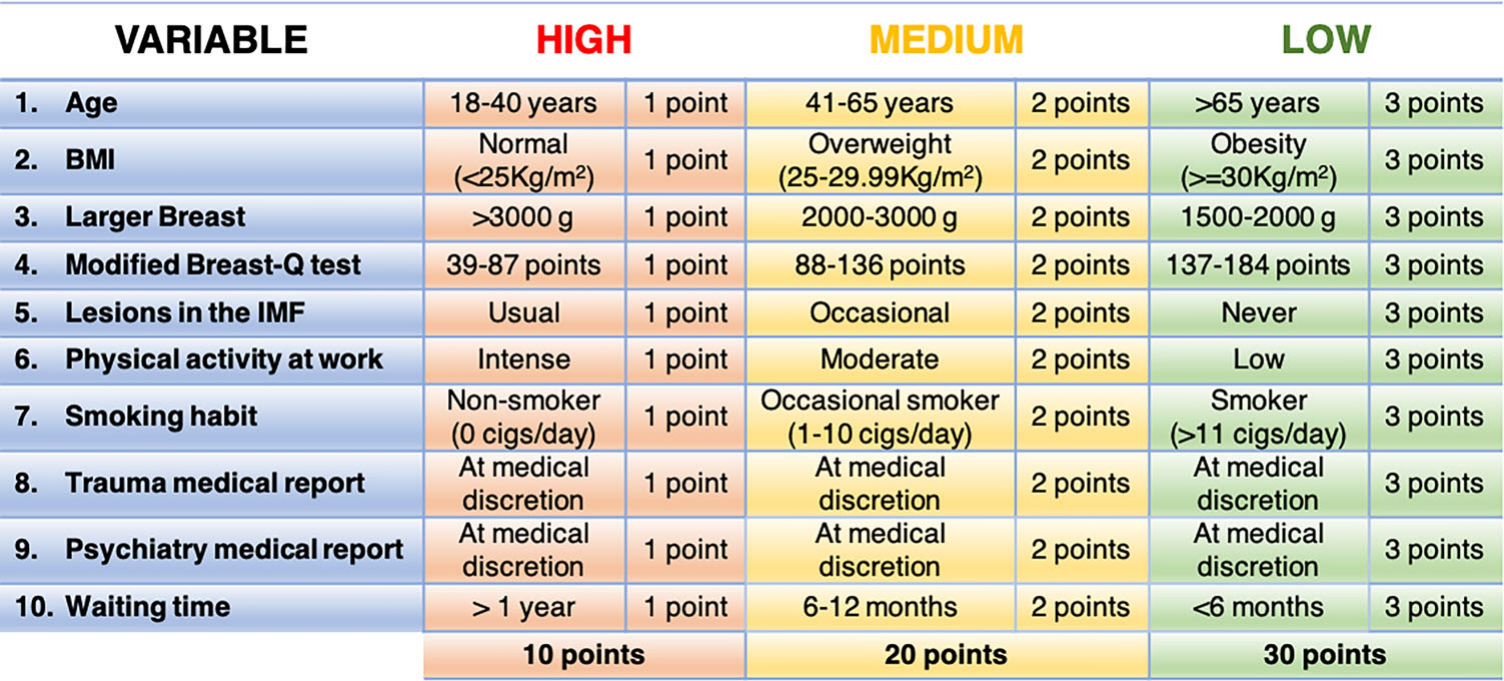
Classification of prioritization levels for each variable in GPS

Finally, the sum of all these variables will compose the final preference score. To facilitate this process, a numerical score of 1, 2, or 3 points will be awarded to the high, medium, and low levels, respectively. The minimum score is 10 points, while the maximum is 30 points. Thus, the lower the score, the higher the priority on the surgical list (Fig. 1).

**Fig. 2:**
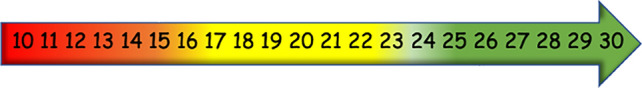
Visual scale for GPS (Red: higher preference score; Yellow: medium preference score; Green: lower preference score)

To further simplify, we have developed a visual scale, as shown in Fig. 2.

Three categories have been established: women scoring 10–16 points will be the top priority candidates for elective surgery, those scoring 17–23 points will be the next in line, and patients with a score between 24 and 30 points will have the lowest priority for surgery.

In cases of equal total scores, the BREAST-Q variable will serve as the determinant for establishing priority, as it is the measure through which patients quantify the impact of the pathology on their quality of life. If patients have identical total and BREAST-Q scores, waiting time will be used as the deciding factor.

The proposed GPS does not seek to extend or modify the established surgical waiting periods but rather to optimize the allocation of available resources within the current system. This model ensures that prioritization is based on clinical need and patient well-being, without altering the overall structure of waiting lists.

### Statistical Analysis

Data were analyzed using descriptive statistics for patient characteristics, calculating mean and standard deviation for quantitative variables. To evaluate the effectiveness of the Gigantomastia Preference Score (GPS), Spearman's Rank Correlation assessed the correlation between original and new rankings, while the Wilcoxon Signed-Rank Test identified significant changes in patient positions. ANOVA and Chi-Square tests were used to examine differences and associations between patient characteristics and priority levels. Statistical significance was set at *p* < 0.05. All analyses were performed using Python 3.8 with pandas, scipy, and sklearn libraries.

### Ethical Approval

This study does not contain any study with human participants. Informed consent does not apply.

## Results

Once the score was defined, we proceeded to analyze the variables recorded in the pre-existing waiting list (WL). Table 3 shows that these patients fall within the medium or high priority levels for each quantitative variable studied, whereas Table 4 specifically shows the percentage of patients at each priority level for each variable based on the total waiting list.

**Table 3:** Clinical and demographic variables

Variable	Mean value (standard deviation)	Priority Level
Age	43.2 years (SD = 13.94)	Medium
BMI	34.61 kg/m^2^ (SD = 6.62)	High
Weight of largest breast	2850 grams (SD = 1644.25)	Medium
Modified BREAST-Q score	74.72 (SD = 19.1)	High
Average waiting time	1545.98 days (SD = 964.13)	High

Data are shown for quantitative variables

**Table 4:** Percentage of patients at each priority level for each variable based on the total waiting list

PL	Age	BMI	LB	BQM	WT	IMFL	PA	SH	TR	PR
High	45,7	12,4	17,9	48	87,32	32	36	72	14	12
Medium	44,2	7,5	42,1	52	12,68	60	32	4	32	33
Low	10,1	80,1	40	0	0	8	32	24	54	55

*PL* Priority level, *LB* Largest breast, *BQM* Breast Q modified, *WT* Waiting time, *IMFL* Inframammary fold lesions, *PA* Physical activity, *SH* Smoking habit, *TR* Trauma report, *PR* Physical report

As observed, the majority of patients (87.6%) were classified as having obesity or being overweight. Additionally, 60% occasionally and 32% regularly experienced IMF lesions. Most patients (64%) had sedentary jobs or were unemployed, and 28% were smokers. Nearly half were classified in the high and medium priority levels for physical and psychosocial impact.

Patients were reorganized on the waiting list using the newly defined score. To assess the effectiveness of the GPS, Spearman's Rank Correlation and Wilcoxon Signed-Rank Test were applied, showing a moderate to strong positive correlation (r = 0.5679, *p* = 1.38e-19) and significant changes in patient positions (statistic = 3485.0, *p* = 8.44e-21). Additionally, ANOVA and Chi-Square tests (Tables 5 and 6) revealed significant differences in patient characteristics across priority levels.

**Table 5:** ANOVA results

Variable	F-statistic	*p*-value
Age	0,0142	0,9859
BMI	5,2084	0,0141*
Largest breast weight	63,1547	7,65E-10**
Modified BREAST-Q score	1,5612	0,2323
Waiting time	0,3239	0,7267

^*^Statistical significance

^**^Highly statistical significance

**Table 6:** Chi-square test results

Variable	Chi-square	*p*-value	Degrees of freedom
IMFL	8,952546	0,062297	4
PA	8,150077	0,086233	4
SH	3,16358	0,530833	4
Trauma report	18,6942	0,000902*	4
Psychiatry report	18,6942	0,000902*	4

^*^Statistical significance

## Discussion

Extended surgical WL pose significant challenges due to varying prioritization criteria across specialties and resource limitations at different facilities. Curtis et al. [39] emphasize the importance of equitable prioritization systems, noting inconsistencies in Australia's current guidelines, such as the lack of specific guidelines, leading to variability in patient access and outcomes. Adopting tools from New Zealand and Canada, which consider clinical and psychosocial factors, could improve patient outcomes and resource use. This approach has shown varied patient survival outcomes, such as in liver transplants, where efficient prioritization optimizes resource allocation and improves survival rates [40].

Reordering WLs may reduce comorbidities, as evidenced by Kelly-Pettersson et al., who found longer waits increase adverse events in hip fracture patients [41]. Dyrstad et al. [42] highlight the economic burden of sickness absence due to extended waiting times. Reorganizing surgical WLs can mitigate these costs by reducing wait times, optimizing resources and even preventing work absences due to illness [43].

Global efforts aim to develop universal prioritization tools considering clinical, functional, and social impacts [37, 44]. Such tools enhance transparency and ensure timely surgery for those in need, as demonstrated in bariatric surgery prioritization tools by Casimiro-Pérez et al., aligning with our GPS approach [45].

At our center, gigantomastia patients wait an average of 4.24 years for surgery, worsening conditions and escalating costs. Studies by Lux et al. [46] and Jud et al. [47] underscore the high costs and limited effectiveness of non-surgical treatments. Our patient demographics align with broader trends, with an average age of 43.2 years and a BMI of 34.61 kg/m^2^ [48, 49].

Upon implementing this score, the statistical analysis showed a moderate to strong positive correlation (r = 0.5679, *p*-value < 0.001) between the original and new waiting list rankings, suggesting a substantial reordering of the list. Statistical analyses showed a strong correlation (r = 0.5679, *p* < 0.001) between original and new rankings, indicating significant changes in patient positions with the GPS. ANOVA results highlighted significant differences in BMI and breast weight across priority levels, justifying their inclusion as critical parameters. Chi-Square tests confirmed significant associations between priority levels and Trauma and Psychiatry reports, underscoring the importance of comprehensive assessments [50, 51].

The GPS aims to address the limitations of traditional waiting lists by ensuring that patient prioritization is guided by objective and clinically relevant criteria. Prioritization based on symptom severity is already a well-established practice in various surgical and medical specialties, including bariatric surgery and organ transplantation. While ethical concerns regarding prioritization exist, particularly in resource-limited systems, our model does not seek to displace patients arbitrarily but rather to optimize surgical scheduling within the constraints of current healthcare structures. Given that long waiting times remain a persistent issue in many public health systems, an evidence-based prioritization model represents a pragmatic approach to maximizing patient well-being while awaiting broader systemic reforms.

### Limitations

The single-center sample may limit generalizability. However, this approach allowed detailed assessment in a specific clinical context. Trauma and psychiatry reports can be subjective, affecting consistency, but ensure comprehensive evaluation. The study didn't assess long-term outcomes, but the immediate impact on patient prioritization was significant, paving the way for future studies.

## Conclusions

The implementation of the GPS significantly reorganized the surgical waiting list, ensuring that prioritization aligns with clinical need and patient-reported quality of life outcomes. BMI, largest breast weight, and specialist reports were key factors. While this study represents an initial institutional evaluation, further research is necessary to validate its applicability in broader healthcare settings. The integration of similar prioritization frameworks in other medical specialties supports the relevance of this approach, reinforcing the importance of refining waitlist management to enhance patient care and resource optimization.

## Data Availability

Data will be provided upon appropriate request
